# Native mechano-regulative matrix properties stabilize alternans dynamics and reduce spiral wave stabilization in cardiac tissue

**DOI:** 10.3389/fnetp.2024.1443156

**Published:** 2024-09-24

**Authors:** Julia Erhardt, Sebastian Ludwig, Judith Brock, Marcel Hörning

**Affiliations:** Institute of Biomaterials and Biomolecular Systems, University of Stuttgart, Stuttgart, Germany

**Keywords:** alternans, spiral waves, cardiomyocytes, mechano-regulation, calcium transients, mechanical contraction, excitation, pattern formation

## Abstract

The stability of wave conduction in the heart is strongly related to the proper interplay between the electrophysiological activation and mechanical contraction of myocytes and extracellular matrix (ECM) properties. In this study, we statistically compare bioengineered cardiac tissues cultured on soft hydrogels ( E≃12 kPa) and rigid glass substrates by focusing on the critical threshold of alternans, network-physiological tissue properties, and the formation of stable spiral waves that manifest after wave breakups. For the classification of wave dynamics, we use an improved signal oversampling technique and introduce simple probability maps to identify and visualize spatially concordant and discordant alternans as V- and X-shaped probability distributions. We found that cardiac tissues cultured on ECM-mimicking soft hydrogels show a lower variability of the calcium transient durations among cells in the tissue. This lowers the likelihood of forming stable spiral waves because of the larger dynamical range that tissues can be stably entrained with to form alternans and larger spatial spiral tip movement that increases the chance of self-termination on the tissue boundary. Conclusively, we show that a dysfunction in the excitation–contraction coupling dynamics facilitates life-threatening arrhythmic states such as spiral waves and, thus, highlights the importance of the network-physiological interplay between contractile myocytes and the ECM.

## 1 Introduction

When the heart’s normal rhythm is out of synchronization, the heart can beat too fast or too slow. Although this can be harmless and just the result of excessive coffee or stress, it can also have life-threatening consequences, causing fibrillation, a stroke, or heart failure (Adam et al., 1984; Konta et al., 1990). During the contraction of a healthy heart, electrical signals are usually transferred between the individual cardiomyocytes in a synchronized way (Gilbert et al., 2020). This process is essentially regulated by calcium (Ca^2+^), which is responsible for the link between the electrical activation (excitation) and the mechanical contraction, known as excitation–contraction coupling (López-López et al., 1995; Bers, 2002; Pfeiffer et al., 2014). If a malfunction in the calcium dynamics occurs, heart rhythm disorders are one possible result (Pfeiffer et al., 2014). Since these disorders affect many people worldwide, scientists try to investigate the underlying causes.

One field of investigation focuses on alternans, a precursor of cardiac arrhythmia. Alternans appears as heartbeats that alternate between strong and weak despite a constant heart rate (Mitchell et al., 1963; Sipido, 2004). At a cellular level, alternans occurs as beat-to-beat alternations in the contraction amplitude (mechanical alternans), action potential (AP) duration (electrical alternans), or intracellular calcium transient (Ca^2+^ alternans) (Edwards and Blatter, 2014; Euler, 1999). It is often found in patients with heart diseases, such as myocardial infarction (Ikeda et al., 2000), and can be triggered by factors such as ischemia (Konta et al., 1990; Dilly and Lab, 1988), an elevated heart rate (Schweigmann et al., 2014), or reduced coronary blood flow due to occlusion Green (1935). Tissue culture experiments reveal two different types: spatially concordant alternans (SCA) and spatially discordant alternans (SDA). In the first type, the whole tissue alternates in phase, whereas the latter can have regions that alternate out of phase (Gizzi et al., 2013; Garfinkel, 2007). Both alternans types are induced by an increase in the heart rate, where SCA appears first, followed by SDA at higher rates (Pastore et al., 1999). If the heart frequency increases even further, SDA gets unstable and consequently initiates a wave break, which can lead to arrhythmic dynamics in the form of spiral waves (Pastore et al., 1999; Karma and Gilmour, 2007). These spiral waves are problematic because they suppress normal heart waves and may lead to fibrillation, i.e., chaotic wave dynamics (Karma and Gilmour, 2007). Regarding the onset of alternans, particularly SDA, a relation to the tissue ultrastructure has been identified (Loppini et al., 2022). A high level of spatial heterogeneity between individual cardiac cells promotes SDA formation already at slower heart frequencies (Loppini et al., 2022; Krogh-Madsen and Christini, 2007).

When conducting *in vitro* experiments, high-frequency electrical pacing represents one commonly used approach to induce contraction in cardiomyocytes and can, therefore, be used to trigger alternans in cell culture or perfused hearts. Studies based on this procedure found that the formation of alternans can be promoted by hypothermia (Gizzi et al., 2017; Loppini et al., 2021), the tissue’s ultrastructure (Loppini et al., 2022), the tissue composition (Kohl and Gourdie, 2014; Bowers et al., 2010), or the substrate rigidity (Hörning et al., 2012a; Hegyi et al., 2021). Considering the rigidity, cardiac cells are more likely to exhibit alternans under high-frequency pacing when cultivated on a substrate that corresponds to the natural rigidity of cardiomyocytes, i.e., 12 kPa (Hörning et al., 2012a; 2013). Similarly, cardiomyocytes embedded in 3D viscoelastic hydrogels, which impose the afterload from vascular resistance during contraction, exhibit both electrical and Ca^2+^ alternans (Hegyi et al., 2021).

As these last two examples illustrate, cells can actively perceive their surroundings and then react to chemical and mechanical changes in their environment, a process known as mechanosensing. This mechanosensing is closely intertwined with cues of the extracellular matrix (ECM), including features like structure proteins, rigidity, or topology, which together function as a network that aids cells in their adhesion, proliferation, migration, and differentiation (Adams and Watt, 1993; Bornstein and Sage, 2002; Missirlis et al., 2022). With regard to mechanical rigidity, often called the E-modulus, cells respond to the forces exerted by a substrate and adapt to the ECM provided (Hörning et al., 2012a; Engler et al., 2004). During *in vitro* experiments, the ECM stiffness is often embodied by hydrogels with an adjustable elasticity. Previous studies reveal a correlation between rigidity and the formation of cell focal adhesion; the stiffer a substrate, the more adhesion sites are expressed, resulting in stronger cell adhesion (Balaban et al., 2001). Additionally, substrates with a higher E-modulus induce changes in cell morphology, i.e., cell area and shape (Engler et al., 2004). Considering migration, cells prefer stiffer regions of hydrogels with a rigidity gradient (Lo et al., 2000; Kidoaki and Matsuda, 2008) and migrate toward an interface when a soft hydrogel with encapsulated cells is placed on a rigid surface (Wei et al., 2020).

In a similar way, the ECM proteins also have an influence on cellular reactions (Bornstein and Sage, 2002). Usually, hydrogels are functionalized with structure proteins, such as collagen or fibronectin, to support cell adhesion (Engler et al., 2004; Missirlis et al., 2022). Hence, a combination of both ECM properties takes place in many studies, and the results indicate that mechanosensing is not only dependent on the viscoelastic properties but also on the binding strength of the surface proteins (Missirlis et al., 2022). Along these lines, we analyzed the morphology and proliferation of myoblasts on different elasticity and fibronectin density compositions in a previous study and found an independent regulation of cell characteristics (Brock et al., 2022). Based on these findings, we use hydrogels that resemble the ECM of cardiomyocytes to observe the formation of alternans in a more natural environment.

In this study, we introduce a simple but effective way to identify and quantify alternans on bioengineered cardiac tissues. We use these quantifiers, which distinguish SCA and SDA with simple V- and X-shaped probability phase map distributions, to investigate the critical threshold of alternans, i.e., the minimum pacing period, before the wave conduction destabilizes and leads to wave breakups. We statically compare tissues cultured on soft hydrogel ( E≃12 kPa) and rigid glass substrates by focusing on the critical threshold of alternans, the restitution and dispersion properties of the tissues, and the formation of stable spirals that manifest after wave breakups. The latter is discussed in terms of the mechano-regulative difference between the substrate conditions by comparing the periodicity of spirals and the corresponding minimum pacing period of planar waves.

## 2 Materials and methods

### 2.1 Glass preparation

Square ( 24×24 mm^2^) and round ( ∅ 22 mm) glass substrates were used and cleaned prior to use, following a modified RCA method (Kern and Puotinen, 1970; Hörning et al., 2017; Brock et al., 2022). In brief, coverslips were successively washed with acetone, ethanol, methanol, and distilled water (MilliQ analytical grade) and sonicated for 3 min in each solution, respectively. A hydrogen peroxide solution (H_2_O: H_2_O_2_: NH_3_ in a 5:1:1 ratio) was added to the coverslips, followed by 3 min of sonication and 30 min of incubation in a 60°C water bath. The coverslips were rinsed 10 times with distilled water and dried at 70°C overnight. The round coverslips were transferred to 5% vinyltrimethoxysilane (Sigma-Aldrich, 235768) in toluene and placed on a shaker in the dark for 18 h at room temperature (RT). Finally, coverslips were washed with acetone, ethanol, and distilled water and baked at 140°C.

### 2.2 Hydrogel preparation

Polyacrylamide (pAAm) hydrogels were prepared from a fresh 1 mL stock solution containing 648.9 μ L distilled water, 174.2 μ L of 40% acrylamide (AAm, Carl Roth, 7748.1), and 154.2 μ L of 2% bis-acrylamide (Carl Roth, 3039.2). This resulted in hydrogels with a Young’s modulus of approximately 12 kPa using a 2% crosslinker ratio and a 1.0 mol ⋅ kg^-1^ total monomer concentration (Brock et al., 2022). A measure of 200 μ L stock solution was prepared with 10% ammonium peroxodisulfate (4.6 μ L, APS, Sigma, A3678) and N,N,N’,N’ -tetramethylethylenediamine (0.3 μ L, TEMED, Carl Roth, 2367.3). From this solution, 40 μ L was sandwiched between a vinyl-silanized round coverslip and a mechanically roughened square coverslip at RT. After 30 min, the square coverslip was removed, and the hydrogels were stored at 37°C in water for at least 48 h prior to use to remove chemical residues.

### 2.3 Mechanical testing

Mechanical measurements of the E-modulus of the hydrogels were performed by nanoindentation using an atomic force microscope (AFM, NanoWizard, JPK Instruments, Berlin, Germany). A silicon nitride cantilever with a nominal spring constant of 0.08 N/m with an attached spherical colloidal probe (CP-PNP-BSG; R = 5 μ m, Olympus Optical) was used. Prior to use, the cantilevers were calibrated, and the spring constant was obtained through thermal noise measurements (Butt et al., 2005). The indentation curves were measured with an approach speed of 1 μ m/s and analyzed using nonlinear least-squares fitting to the Hertz model (Sneddon, 1965); Domke and Radmacher, 1998) with a customized MATLAB (MATLAB, 2023) routine, as follows:

$$F=4ER^{1/2}\cdot {\left[3{\left(1-\nu \right)}^{2}\right]}^{-1}\cdot \delta^{3/2}\text{,}$$ (1)

where F is the applied force, ν=0.5 is the Poisson’s ratio, and δ is the indentation depth (Lin et al., 2006). Statistical significance was ensured by the quantification of the Young’s modulus E at 50 independent indentation sites in two 100×100 μ m^2^ areas for each hydrogel (Brock et al., 2022).

### 2.4 Height determination

For the determination of the hydrogel height, hydrogels were stained with a 0.9 mM rose bengal solution (Sigma, 330000) for 1 h in the dark at RT, followed by incubation in distilled $H_{2}$ O for 1 h in the dark at RT as a washing step. Subsequently, the height was measured using a confocal laser scanning microscope (LSM 980 with Airyscan 2, Carl Zeiss Microscopy GmbH, Jena, Germany) equipped with a 40 × magnification objective lens (LD LCI Plan-Apochromat 40 × /1.2 Imm Korr DIC; Carl Zeiss Microscopy GmbH, Jena, Germany) and a GaAsP-PMT detector. The laser intensity was set to 10% at a 561-nm excitation wavelength. The images were recorded in multiplex CO-8Y mode with a size of 1848×1852 pixels (16 bit) and 241 focal heights ( Δz=1 μ m). The image stacks were acquired and processed using ZEN blue v3.3 software (Carl Zeiss Microscopy GmbH, Jena, Germany). The average intensity signal of each image was calculated and normalized to determine the hydrogel height by measuring the distance between the intensity peaks at the glass-to-gel and gel-to-water interfaces. All data were analyzed using ImageJ (1.54f).

### 2.5 Surface functionalization

Polyacrylamide gels were functionalized with fibronectin from human plasma (Sigma, F2006) using 3,4-dihydroxy-L-phenylalanine (L-DOPA, Sigma-Aldrich, D9628)) before cells were seeded. L-DOPA was prepared at a final concentration of 2 mg/mL in 10 mM Tris buffer (pH 10, Roth, 4855.1), incubated under shaking conditions for 30 min in dark, and sterile-filtrated through a 0.2- μ m filter (Filtropur S0.2, Sarstedt, 83.1826.001). The polyacrylamide hydrogels were washed with Tris buffer, and 250 μ L of the L-DOPA solution was added, incubated in the dark for 30 min, and washed twice with phosphate-buffered saline (PBS). Fibronectin solutions were applied at a density of 0.4 μ g/cm^2^ on glass coverslips and 4.0 μ g/cm^2^ on hydrogels. After the application, the samples were incubated at 37°C for at least 2 h (Brock et al., 2022).

### 2.6 Cardiomyocyte isolation

For the preparation of cardiac tissue sheets, the hearts of 1–3-day-old Wistar rats were isolated. Isolated hearts were cleaned, minced, and enzymatically digested in five cycles using collagenase type I (Thermo Fisher). The isolated cells from the last four cycles were pre-plated for 1 h in plastic dishes to reduce the fraction of fibroblasts (Hörning et al., 2012a; 2017). Cells were plated at a density of 2.6×10 ^5^ cells/cm^2^ in Dulbecco’s modified Eagle medium (DMEM, Gibco, 11885084) supplemented with 10% fetal bovine serum (Gibco, 10270106), 1% penicillin–streptomycin (Gibco, 15140122), and kanamycin sulfate (80 mg/L; Gibco, 11815024). After 24 h, the medium was exchanged to the minimal essential medium (MEM, Gibco, 11095080) supplemented with 10% calf serum (Gibco, 16,170-087), 1% penicillin–streptomycin, kanamycin sulfate (80 mg/L), and cytosine arabinofuranoside (ARA-C, 10 μ M; Sigma-Aldrich, C1768). The latter is a proliferation inhibitor used to maintain a high cardiomyocyte-to-fibroblast ratio in the culture (Hörning et al., 2012b). The cardiac tissues were observed after 4 days of incubation.

### 2.7 Observation

Prior to observation, the contractile cardiac tissue sheets were incubated with 200 μ L of the Ca^2+^-sensitive dye Fluo-8 (8.3 μ M in PBS, AAT Bioquest) at RT in the dark for 1 h. Samples were observed in Tyrode’s solution, containing 136.9 mM NaCl, 1 mM MgCl_2_, 2.7 mM KCl, 1.8 mM CaCl_2_, 0.4 mM NaH_2_PO_4_, and 5.5 mM glucose (Sigma-Aldrich, T2145) with additional 2.7 mM KCl (final concentration of 5.4 mM) and 5 mM HEPES (Roth, 9105.2). The pH level was adjusted to 7.4 using NaOH. Ca^2+^ transient dynamics were observed with a customized microscope set-up (Thorlabs) equipped with a Kinetix sCMOS High-Speed Camera (Photometrics, 140 FPS and Δ x = Δ y = 48 μ m/px after 4×4 binning) and a zoom objective (PlanApoZ 0.5 × /0.125 FWD 114 mm, Carl Zeiss). The tissue samples were observed at RT, if not stated differently. In this case, the tissue samples were observed at 37°C on a customized incubation stage that was heated by two polyimide heat foils (Thermo TECH, 3626108, 23×140 mm) and electronically regulated by a temperature switch (H-Tronic, TSM 125).

### 2.8 Electrical stimulation

Cardiac tissue sheets were electrically stimulated using platinum electrodes ( ∅=0.5 mm, 99.997%, Thermo Fisher Scientific/Alfa Aesar) and a modified version of the MyoPulser stimulator introduced by Ott and Jung (2023). The device was constructed using a motor controller (5–35 V, $I_{\text{max}}$ = 2 A) and a microcontroller (ESP32-S2-WROVER, Espressif) programmed with an Arduino IDE (2.3.2). A power supply unit with a voltage of 12 V and current up to 2 A was used. A customized graphical user interface (GUI) was implemented using software Processing (The Processing Foundation). The device was enclosed in a customized 3D-printed chassis (PRUSA MK3s, Prusa Research), designed using CAD software SolidWorks (Dassault Systèmes). The local stimulation of ±10 V was applied with 10-ms bipolar pulses delivered through 1-mm-spaced electrodes at the right edge of the samples.

### 2.9 Fluorescence staining

Selected samples were washed for 5 min with PBS-EGTA (PBS with 2 mM EGTA, Roth, 3054.2), fixed for 5 min in 4% formaldehyde (in PBS, Thermo Scientific, 175 J60401. AK) in PBS-EGTA with 0.1% Triton X-100 (Roth, 3051.3), and washed twice with ice-cold PBS for 5 min. The samples were blocked for 20 min with 400 μ L of 1% BSA (Sigma, A9418) and 0.1% Triton X-100 in PBS. As a primary antibody, sarcomeric α -actinin (Invitrogen, MA1-22863) was diluted 1:200 in PBS with 1% BSA and 0.1% Triton X-100, and 200 μ L of this solution was added to each sample and incubated for 1 h. The secondary antibody, Alexa Fluor 488 (Invitrogen, A-11001), was diluted 1:200 in PBS with 0.1% Triton X-100. DAPI (1 mg/mL; Sigma-Aldrich, D9542) was added at 1:1000 dilution, and Alexa Fluor 546 phalloidin (200 U/mL in methanol, Invitrogen, A22283) was added at 1:800 dilution. To each sample, 200 μ L of the antibody mixture was added and incubated in the dark for 1 h. The tissue sheets were then washed twice in PBS and permanently fixed to objective slides (Labsolute, 7695002) with ProLong™ Gold Antifade Reagent (Invitrogen, P10144).

### 2.10 Confocal microscopy

The fluorescent-stained tissues were imaged using an Axio Observer Z1/7 spinning-disk confocal microscope with a ×40 objective (Plan-Apochromat 1.4 Oil DIC UV, Zeiss) and an Axiocam 503 Mono CCD Camera (Carl Zeiss Microscopy GmbH) at a resolution of Δx=Δy=0.227 μ m. For the visualization of nuclei (DAPI), α -actinin (Alexa 488), and phalloidin, lasers with 405 nm, 488 nm, and 561 nm wavelengths were used, respectively. The image acquisition was performed using ZEN blue v2.3 software (Carl Zeiss Microscopy GmbH, Jena, Germany). Image compositions of 5×5 lateral tiles with up to 10 focal heights were recorded. The tiled images were stitched and shade-corrected before a final projected image ( 810×760 μ m^2^) was calculated by the maximum intensity. Image post-processing for the final image compositions was performed using ImageJ (1.54f).

### 2.11 Data analysis

Data were analyzed using customized routines in MATLAB (MATLAB, 2023). The recorded movies were subsequently pre-processed by background subtraction, averaged in time (10 frames), and filtered in space with Gaussian blur (10 px) using ImageJ (1.54f) (Loppini et al., 2022).

Restitution properties and conduction velocity (CV) were extracted from individual waves by the detection of the upstroke and maxima of waves. The calcium transient (CT) was analyzed at a normalized calcium intensity of 50% of each individual wave to obtain the calcium transient duration (CTD) and calcium transient interval (CTI). The CV was calculated from a straight line along the direction of wave propagation. A linear regression fit of all extracted locations along that line was used to calculate the CV for each wave.

The normalized CTD maps were calculated by taking advantage of the periodic signal using the signal oversampling technique (Hörning et al., 2017). The periodic signal is stacked by equidistant time intervals, which reduces the noise ratio as the square root of the number of measurements (Uzelac and Fenton, 2015). Hence, the pixel-wise-averaged CTD was computed from the stacked calcium transient ⟨CT⟩ , as follows:

$$\left\langle \text{CT}\right\rangle =\overset{N}{\underset{i}{\sum }}{\text{CT}}_{i}\left[i\cdot \left(T+\tau \right)\right]\text{,}$$ (2)

where ${CT}_{i}$ represents the calcium transients of the individual waves, T is the pacing period, and τ is the offset induced by frame rate inaccuracies of the camera ( τ≤140Hz≃7 ms). From ⟨CT⟩ , the averaged CTD was extracted following the same approach used for the individual waves.The normalized difference between the CTDs of odd and even beats ( Δ CTD) is calculated from the period-2 calcium transient data:

$$\left\langle {\text{CT}}_{2}\right\rangle =\overset{N/2}{\underset{i}{\sum }}{\text{CT}}_{i}\left[2i\cdot \left(T+\tau \right)\right]\text{,}$$ (3)

as

$$\begin{array}{ccc} \Delta \text{CTD} & = & \left|\text{CTD}\left({\left\langle {\text{CT}}_{2}\right\rangle }^{\text{odd}}\right)-\text{CTD}\left({\left\langle {\text{CT}}_{2}\right\rangle }^{\text{even}}\right)\right|\\ & = & \left|{\text{CTD}}^{\text{odd}}-{\text{CTD}}^{\text{even}}\right| \end{array}$$ (4)

for each individual pixel. The normalized CTD is calculated as the mean of ${CTD}^{\text{odd}}$ and ${CTD}^{\text{even}}$ in case of a period-1 CT (no alternans).

## 3 Results

The electromechanical wave conduction stabilizes and alters the offset of alternans initiation when the rigidity of the substrate matches that of the cardiac cells, i.e., Young’s modulus for both the hydrogel and cardiac cells is E≃12 kPa (Hörning et al., 2012a; 2013).

**FIGURE 1:**
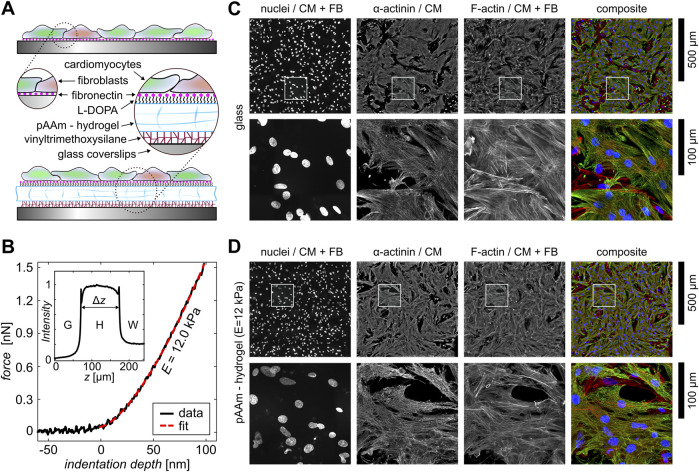
Experimental setup of cardiac monolayers on different substrates. **(A)** Schematic illustration of the different bioengineered cardiac monolayer constructs on glass and hydrogels. **(B)** One exemplary nano-indentation curve measured at the pAAm-hydrogel (black data). The red dashed line shows the corresponding fit. The inset shows a rose-bengal-stained signal intensity profile of a hydrogel. The normalized intensity is shown as a function of the height z . The two peaks mark the transitions from glass (G) to hydrogel (H) and hydrogel (H) to water (W), respectively. The gel height Δz is calculated as the peak-to-peak distance. **(C, D)** Fluorescence staining of cardiac monolayers composed of cardiomyocytes (CMs) and fibroblasts (FBs) on glass and hydrogel. The lower row shows the zoomed-in sections of the white square in the upper images. Nuclei, α -actinin, F-actin, and the composites of these are shown from left to right.

In this study, we introduce a stable *in vitro* platform for cardiac tissues that closely mimics the biological ECM and reliably withstands the strong forces of contractile cardiac cells for the investigation of the electromechanical initiation of alternans. For that, we compare two substrate conditions. On one hand, we use glass substrates that are coated with fibronectin, as glass substrates are still used in most studies that investigate the dynamics of primarily cultured (Entcheva et al., 2000; Hörning et al., 2017) and stem cell-derived cardiac tissues (Klimas et al., 2016; Heinson et al., 2023). On the other hand, we utilize the ECM-mimicking hydrogel platform that has been introduced recently for the study of myocyte proliferation dynamics (Brock et al., 2022). Figure 1A schematically illustrates the difference between both substrates. Although the fibronectin is simply coated on glass and may be influenced by the dynamics of cells, it is covalently bound by L-DOPA to the hydrogel. The hydrogels are bound to the glass substrates using vinyltrimethoxysilane (see Section 2), which reliably withstands the strong contractile forces of the cells during tissue development and subsequent observation. The hydrogels were prepared with a rigidity of E=12.3±1.1 kPa (Equation 1) and an average height of approximately 100 μ m. A typical AFM-recorded indentation curve and a rose-bengal-stained signal intensity profile of the hydrogel are shown in Figure 1B.

Primary cardiac tissue culture is naturally composed of cardiac and fibroblast cells. The fraction of both defines the stability of wave conduction (Petrov et al., 2010; Rother et al., 2015), i.e., a lower likelihood of wave breaks that may lead to spiral waves. So, using the proliferation inhibitor ARA-C, we minimize the number of fibroblasts. Figures 1C, D show large, high-resolution image composites of fluorescent-stained, confluent cardiac tissues on glass and hydrogel. The figures include single-channel images of the cell nuclei, the sarcomere-specific α -actinin, and the F-actin, as well as false-color composites (right panels). The upper rows illustrate the entire images, while the lower rows show zoom-in sections that are marked by white squares in the upper images.

### 3.1 Quantification of alternans

**FIGURE 2:**
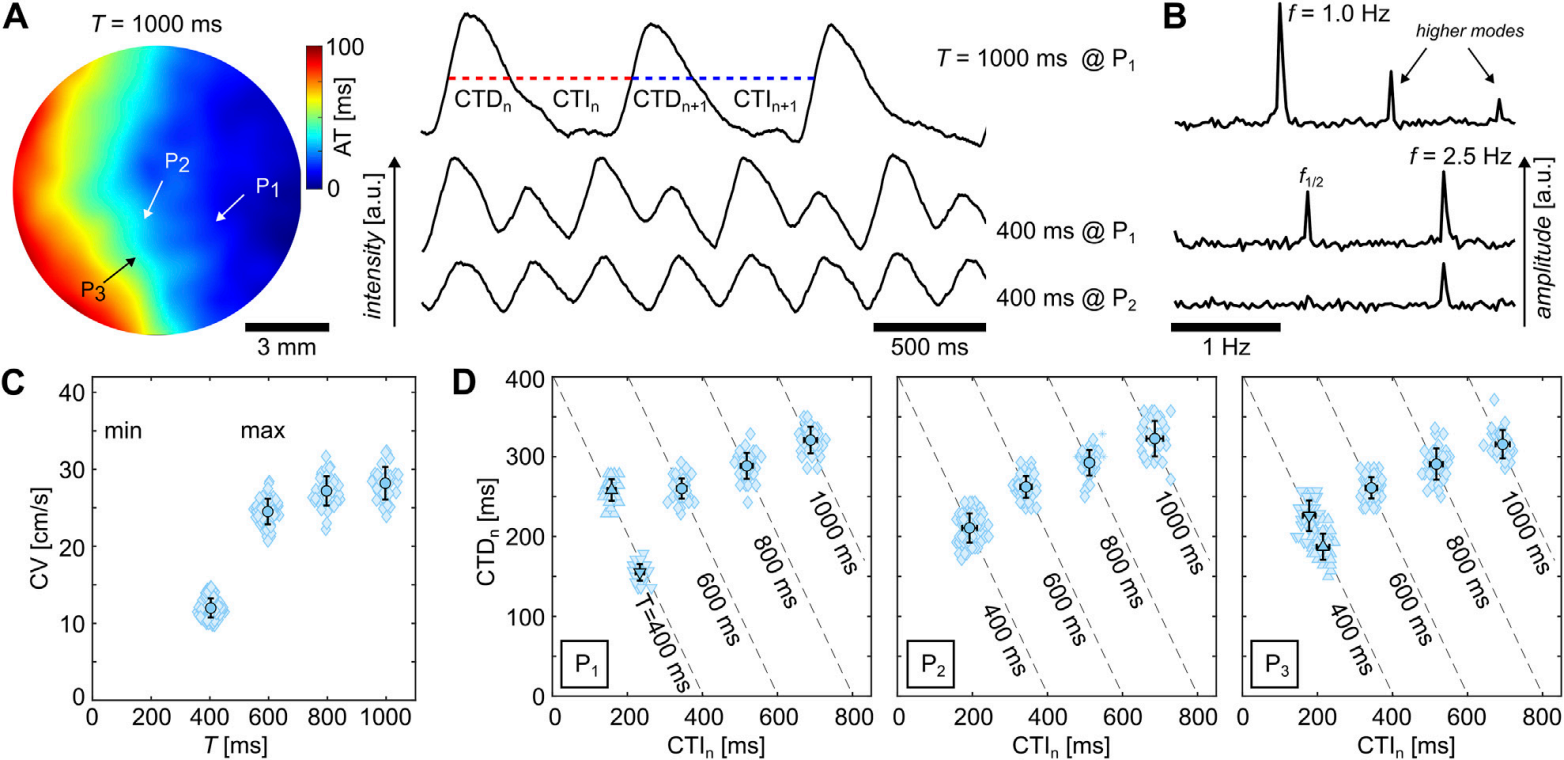
Examples of calcium transient dynamics in cardiac monolayers cultured on glass observed at 37°C. **(A)** Activation time map of a T=1000 ms entrained cardiac tissue. Three Ca^2+^ signals entrained with T=1000 ms and T=400 ms and measured at two independent locations ( $P_{1}$ and $P_{2}$ , 7 × 7 pixels FOV). SDA is observed at T=400 ms, leading to locations with ( $P_{1}$ ) and without ( $P_{2}$ ) alternans. **(B)** Fourier spectra of the three Ca^2+^ signals illustrated in **(A)**. The main frequency f is defined by the pacing period T . The peaks after f are the higher modes ( T=1000 ms), and the presence or absence of a second peak $\left(f_{1/2}\right)$ before f indicates the presence or absence of alternans ( T=400 ms). **(C)** Dispersion curve of the same sample. **(D)** Restitution curves at three different locations $P_{1}$ , $P_{2}$ , and $P_{3}$ . **(C, D)** The light blue data points correspond to individual calcium waves, and the solid data points correspond to the mean value and standard deviation. Upward and downward triangles illustrate the presence of alternans with the corresponding phase. The asterisk markers depict outliers.

Alternans can occur in high-frequency-entrained (Gizzi et al., 2013) and freely rotating and heterogeneity-pinned spiral waves (Hörning et al., 2017). At low wave frequencies, no alternans is observed as the electrophysiological dynamics recover to their resting state and no wave-to-wave interaction takes place. Higher frequencies, however, can lead to alternans. Figure 2A shows a typical example of glass observed at 37°C. The upper two signals show CTs at pacing periods of T=1000 ms and T=400 ms at the same location $P_{1}$ within the tissue. The latter shows a period-2 alternans, i.e., different CTs are formed for the even and odd waves. Although alternans is observed at $P_{1}$ , no alternans is observed at a different location $P_{2}$ (see lower signal). This indicates that alternans is spatially restricted within this tissue.For the determination of the alternans rhythms, Fourier transformation imaging (FFI) can be used (Hörning et al., 2017) as it can also identify high-order alternans, such as period-4 rhythms, which can be easily visualized without the need for spatiotemporal filters (Loppini et al., 2022). Figure 2B exemplarily shows a single-pixel trace from the FFI analysis of the CTs shown in Figure 2A. In the case of a single period, i.e., no alternans, only the main peak at $f=T^{-1}$ and its higher modes are present. In contrast, in the case of alternans, a secondary peak at $f_{1/2}={\left(2T\right)}^{-1}$ forms that refers to the periodic repetition of the two repetitive CTs.

Alternans can be observed either exclusively or in combination in the APs, CTs, and CVs (Edwards and Blatter, 2014; Euler, 1999). In the case of the example illustrated in Figure 2, the CV does not exhibit alternans. Figures 2C, D show the restitution and dispersion curves of the sample observed at T=400 ms, 600 ms, 800 ms, and 1000 ms. The CV is exemplarily measured at the center of the tissue. The dispersion curves of three locations are shown by the calcium transient durations ( ${CTD}_{n}$ ) *versus* the calcium transient intervals ( ${CTI}_{n}$ ) of the individual CTs with transparent blue data points, where the index n refers to the wave number (see also Figure 2A). The mean values are shown as solid blue data points. The upward and downward triangles refer to the odd and even CTs in case of period-2 alternans, as shown at $P_{1}$ and $P_{3}$ for the stimulation period of T=400 . Contrarily, round data points are shown in cases where no alternans is observed. Although only three locations are shown, the data indicate the occurrence of spatially discordant alternans (SDA), which means that different regions in the sample alternate out-of-phase. This is visualized by a change in the positions of the upward and downward triangles. At $P_{1}$ , odd waves (upward triangles) have the longer CTD, while it is *vice versa* at $P_{3}$ . Thus, the dispersion curve at $P_{2}$ indicates a nodal line, which acts as an interface between the out-of-phase-alternating neighboring regions within a tissue.

**FIGURE 3:**
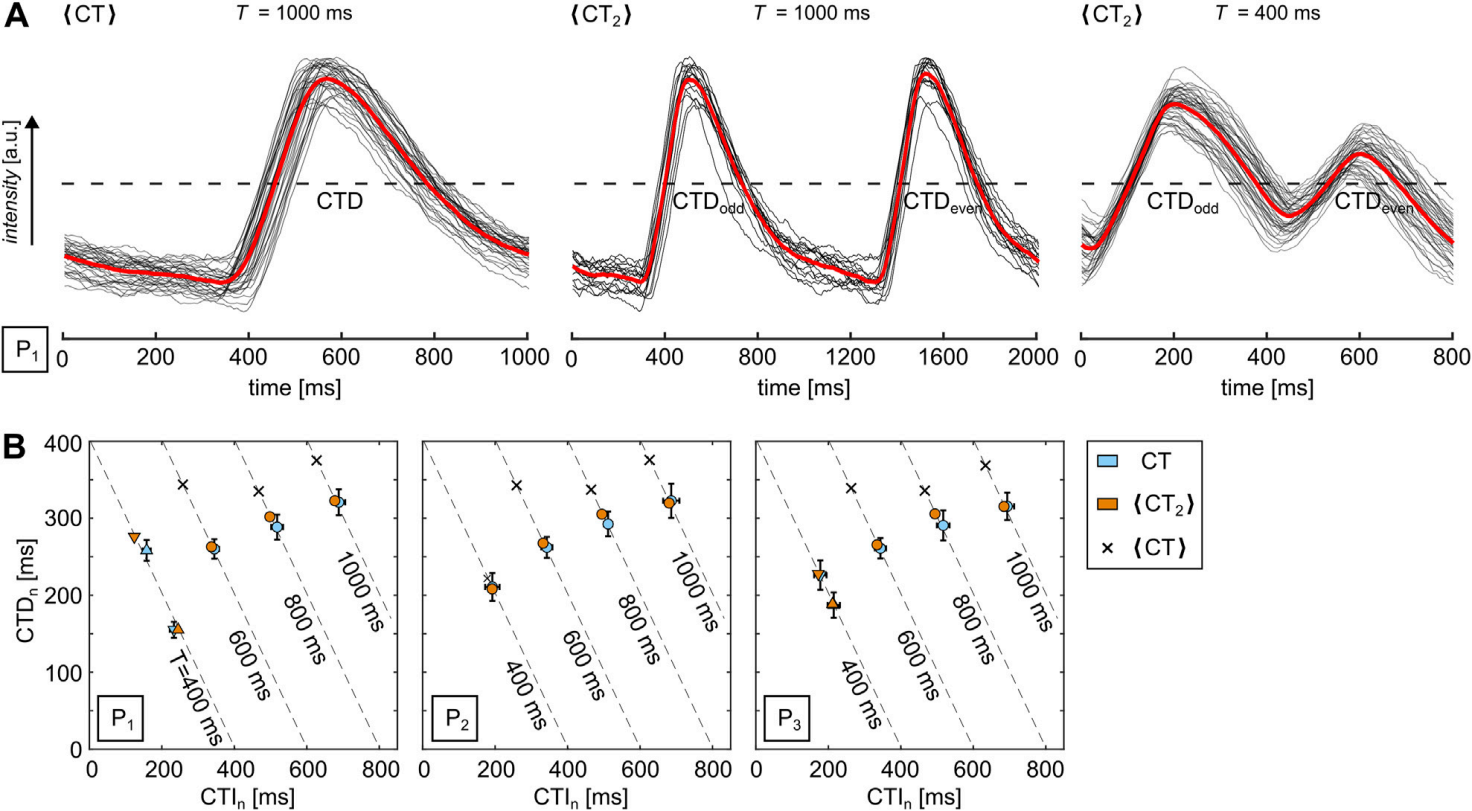
Quantification of alternans via signal oversampling. **(A)** Examples of normalized calcium transience (red lines) for T=1000 ms and T=400 ms. The black lines show the individually stacked CTs. The three panels show ⟨CT⟩ for T=1000 ms, $\left\langle {\text{CT}}_{2}\right\rangle$ for T=1000 ms, and $\left\langle {\text{CT}}_{2}\right\rangle$ for T=400 ms that exhibits alternans. The black dashed lines indicate the intensity levels at 50%. **(B)** Comparison of the normalized calcium transience quantification methods for the single CT extraction, as shown in Figure 2D (blue circles), ⟨CT⟩ (black cross), and $\left\langle {\text{CT}}_{2}\right\rangle$ (orange circles).

In order to visually confirm the presence of SDA, every pixel of the recorded movie generally needs to be analyzed, as shown in Figure 2D, and the mean values have to be pixel-wise recomposed to a single image. However, this might lead to problems with noisy data or alternatively imply the need for stronger spatiotemporal filters since every individual CT must be identified. So, in this study, we use a signal oversampling technique (Hörning et al., 2017) to generate normalized CTD maps as it reduces the noise ratio by the square root of the number of measurements (Uzelac and Fenton, 2015). The repetitive wave patterns are stacked by an equidistant time interval, T in a periodically stimulated tissue, to create a normalized ⟨CT⟩ (see Equation 2). From the ⟨CT⟩ , CTD and CTI can be similarly extracted as for single CTs. The first panel of Figure 3A shows the noise reduced ⟨CT⟩ (red solid line) that is obtained from the stacked individual CTs (black lines) for T=1000 ms.

However, a disadvantage of this approach is the accumulating time shift δ , especially for lower-recorded frame rates. In this study, the time shift is up to approximately δ≃3.6 ms=τ/2 for a frame rate of 140 s^-1^ (τ≃7.2ms) as a single frame does not necessarily align with T . It might round up or down to a frame more or less, leading to a δ of up to half of the frame rate. Therefore, δ leads to a continuous drift of CTs and, thus, to an increased normalized CTD. This increase is linearly proportional to the number of CTs. To overcome this issue, we analyzed the data over an interval of 2T (Equation 3) instead of T , which involves analyzing odd and even CTs independently. This approach is necessary for the analysis of period-2 alternans (see Figure 3A). We refer to this analysis as $\left\langle {\text{CT}}_{2}\right\rangle$ , which leads to a reduced effective drift as the number of analyzed wavelets and δ reduce by a factor of two. Figure 3B illustrates the comparison of the extracted CTD between the three different approaches, i.e., CT, ⟨CT⟩ , and $\left\langle {\text{CT}}_{2}\right\rangle$ , for the data shown in Figure 2. While the data show a large deviation for ⟨CT⟩ (black crosses) from CT, i.e., the average of the individual CTs (blue circles), $\left\langle {\text{CT}}_{2}\right\rangle$ (orange circles) agrees very well with CT.

### 3.2 Spatial quantification of alternans

**FIGURE 4:**
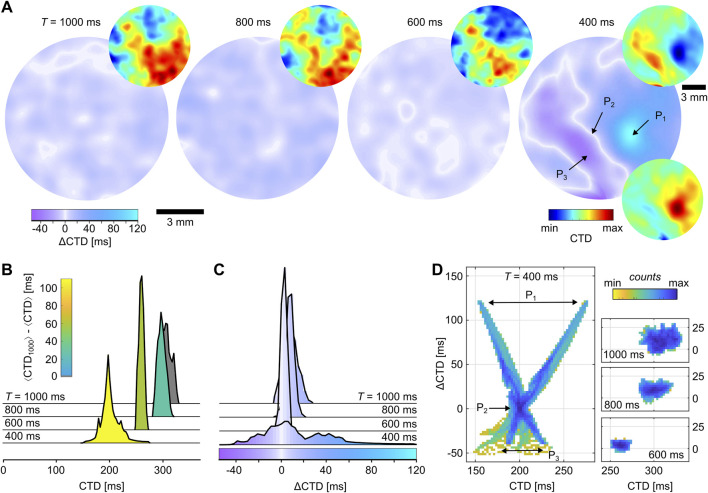
Calcium transient dynamics in cardiac monolayers cultured on glass. **(A)** Normalized CTD (small) and Δ CTD maps (large) for different T . For T=400, an example of SDA is shown. The locations $P_{x}$ correspond to the data shown in Figure 2. The two CTD maps correspond to the even and odd waves. **(B, C)** CTD and Δ CTD distributions for the four examples that are shown in **(A)**. The color code in **(B)** represents the difference between ⟨CTD⟩ and ⟨CTD⟩ observed at T=1000 . The extrema [min, max] of the four CTD distributions used to normalize the CTDs in **(A)** are [284 ms, 331 ms], [283 ms, 319 ms], [249 ms, 272 ms], and [137 ms, 276 ms]. **(D)** Probability phase maps of CTD and Δ CTD. $P_{1}$ , $P_{2}$ , and $P_{3}$ indicate the data of the positions shown in Figure 2 observed at T=400 ms.

For the spatial quantification of alternans, the pixel-wise analysis of $\left\langle {\text{CT}}_{2}\right\rangle$ is used to obtain the normalized CTD and Δ CTD maps for different T . Δ CTD is calculated from the difference of the subsequential odd and even CTDs (see Equation 4). Figure 4A shows the normalized CTD (small circles) and Δ CTD maps (large circles) for the tissue with the same pacing periods that were used in Figure 2. For larger pacing periods ( T≥600 ms), the waves have a constant CTD and, thus, lead to values of ΔCTD≃0 ms. Contrarily, SDA is shown in the case of alternans ( T=400 ms). The Δ CTD map shows nodal lines (white lines, ΔCTD≃0 ms) that spatially separate the two out-of-phase-oscillating period-2 alternans regimes in negative and positive Δ CTDs. The two corresponding normalized CTD maps are shown above and below the Δ CTD map. Interestingly, the CTD map for T=600 ms resembles the CTD map (above) at T=400 ms. So, despite the large difference in T , the spatial distribution of CTDs is already measurable at lower pacing periods that do not lead to alternans. Figures 4B, C show the distributions of CTD and Δ CTD. Both show similar trends with the decrease in T ; for example, the distributions become narrower, and with the occurrence of alternans, a much broader distribution is observed. The color coding in Figure 4B describes the difference from the largest observed pacing period, which is T=1000 ms in this study, and the color coding in Figure 4C corresponds to the same scheme used in Figure 4A. The largest alternation of CTDs is observed at location $P_{1}$ with ΔCTD≃120 ms.

Although the normalized CTD and Δ CTD maps can be used to extract a lot of information and quantify the spatial organization of dynamics, they do not necessarily give clear evidence for the occurrence of alternans. This is because nodal lines might also be identifiable at T=600 ms (see Figure 4A), where small rings of white lines are visible that might be—here wrongly—interpreted as nodal lines. Therefore, a clear, unequivocal, non-interpretable way to identify alternans is necessary. By introducing probability phase maps of CTDs and Δ CTDs from the entire tissue, unique patterns can define the difference in SCA, SDA, and normal wave conduction. Figure 4D shows probability phase maps of CTD and Δ CTD. Although larger pacing periods ( T≥600 ms) show spatially restricted distributions that narrow with the decrease in T , SDA shows an unequivocally X-shaped distribution. The latter naturally assembles due to positive and negative values that correspond to larger CTDs. This indicates that the larger the CTD, the larger or smaller the Δ CTD in the case of SDA. So, despite the small white rings that might be interpretable as nodal lines at T=600 ms, alternans is observed in these four examples only for T=400 ms. Next, by decreasing the incremental steps of the pacing period, we investigate the initiation and transition of alternans from SCA to SDA. Although not shown yet, SCA should lead to a ‘V’-shaped probability phase map because Δ CTD values are only positive due to the missing phase shift.

### 3.3 Initiation and transition of alternans

**FIGURE 5:**
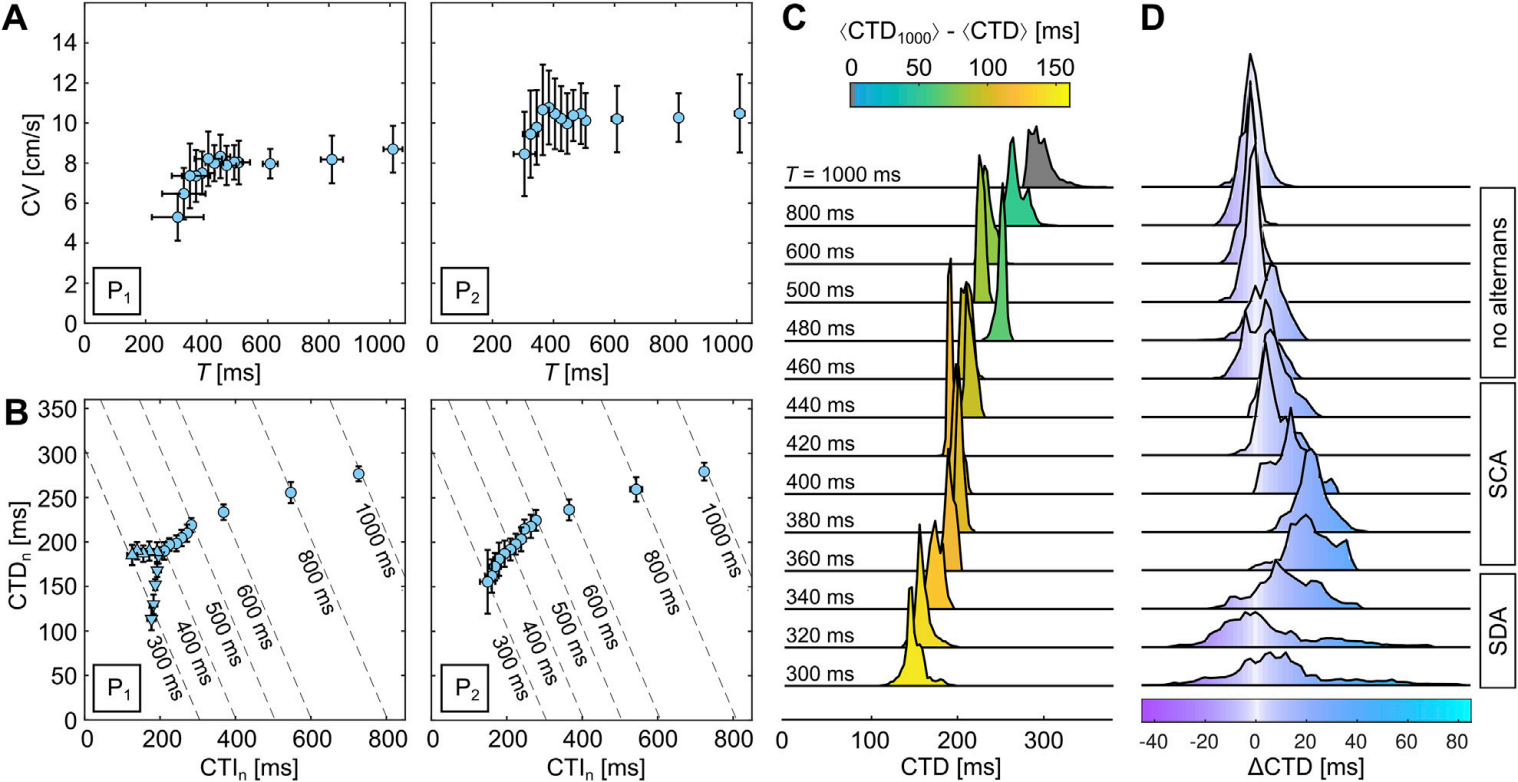
Quantification of calcium transient alternans in cardiac monolayers cultured on hydrogels. **(A, B)** Dispersion and restitution curves for two locations, $P_{1}$ and $P_{2}$ , within a tissue. $P_{1}$ shows period-2 alternans for approximately T=320 ms, and $P_{2}$ does not show alternans. **(C, D)** Respective distributions for CTD and Δ CTD. The color code in **(C)** represents the difference of ⟨CTD⟩ to the one at T=1000 . The column on the right side in **(D)** indicates the regimes (pacing periods) of the absence of alternans, SCA, and SDA.

Following the same approach as shown for the cardiac monolayers cultured on glass, we now investigate exemplarily a cardiac monolayer that is cultured on hydrogel (see Figure 1). Furthermore, we change the observation setup to RT as hypothermia promotes the formation of alternans (Gizzi et al., 2017; Crispino et al., 2024). For identifying the initiation and transition of alternans, we increased the number of stimulation periods in the range of T=500 ms to T=300 ms in steps of ΔT=20 ms and below T=300 ms in steps of ΔT=10 .This approach is used because alternans initiation is expected in tissues cultured on soft substrates within this regime (Hörning et al., 2012a). Figures 5A, B show the CV and ${CTD}_{n}$ for two locations, $P_{1}$ and $P_{2}$ , exemplarily. Only at $P_{1}$ , period 2-alternans is observed in the CTD at T=420 ms, indicated by the upward and downward triangles, as shown in Figure 2. No alternans is observed for the CV (Figure 1A). The distributions of CTDs obtained from the $\left\langle {\text{CT}}_{2}\right\rangle$ analysis (Equation 3) show a linear decrease in the average CTDs (Figure 5C, ⟨CTD⟩ ). A few slight deviations from the linear trend are observed, e.g., at T=480 ms and 420 ms, which might be caused by longer delays between the individual observations as the calcium storages in these samples might more slowly than in others. Generally, the time delay between observations is kept at approximately less than 10 seconds. Figure 5D shows the corresponding distributions of Δ CTD. For larger T , the ⟨CTD⟩ values are approximately 0. From T=440 ms, a shift toward positive Δ CTD distributions is observed, which indicates the occurrence of SCA, and from T=340 ms, a backward shift and broadening of the distributions are observed, which indicates the occurrence of SDA.

**FIGURE 6:**
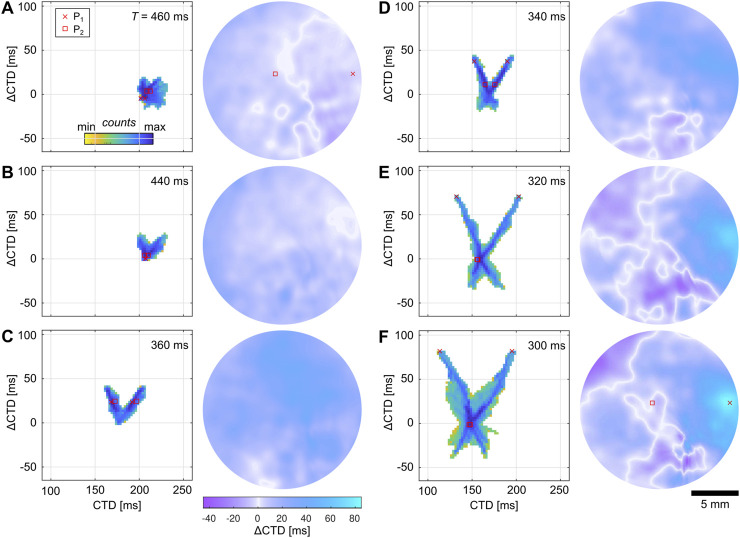
Spatial quantification of calcium transient alternans in cardiac monolayers cultured on hydrogels. **(A–F)** Probability phase maps (left) and Δ CTD maps (right) for different stimulation periods T . **(A, B)** Transition from the absence of alternans ( T=460 ms) to SCA ( T=440 ms), i.e., from a round to a V-shaped distribution. **(C, D)** Transition from SCA ( T=360 ms) to SDA ( T=340 ms), i.e., from a V-shaped to an X-shaped distribution. **(E, F)** Transition from a stable SDA ( T=320 ms) to a more unstable SDA ( T=300 ms) distribution before the initiation of spiral wave breaks at T=290 ms. The red crosses and squares mark the CTD and Δ CTD of the locations $P_{1}$ and $P_{2}$ (see Figures 5A, B).

The transitions to SCA and SDA can be visualized using probability phase maps. Figures 6A, B exemplify the transition of alternans to SCA, i.e., from a spherical to a V-shaped distribution. Although the Δ CTD map in Figure 6A still shows areas close to ΔCTD≃0 ms with both positive and negative areas of Δ CTDs, the negative Δ CTDs are significantly reduced, leading to an almost perfectly V-shaped distribution, thus indicating SCA. This continues until T=360 ms, where the size of the V-shaped distribution is the largest, indicating the lowest pacing period where SCA is observed. At T=340 ms, an X-shaped distribution is formed, and the reappearance of nodal lines can be seen (Figure 6D). This indicates that SDA occurs. As T decreases, the size of the X-shaped distribution and the complexity of the nodal line patterns (Figure 6E, T=320 ms) increase until a critical period is reached, at which point the X-shaped distribution starts to collapse (Figure 6F, T=300 ms). Hence, T=300 ms is the lowest pacing period where waves can propagate through the tissue without experiencing local conduction blocks. Single locations within the probability maps, $P_{1}$ and $P_{2}$ , are marked as red crosses and squares, respectively. Additionally, the locations are marked in the Δ CTD maps in Figures 6A, F. An overview of all probability phase maps observed for that sample can be seen in Supplementary Figure S1 (see Supplementary Material). The most pronounced SCA is observed for T=300 ms, where almost the entire tissue is oscillating with SCA.

### 3.4 Transition to spiral wave initiation

**FIGURE 7:**
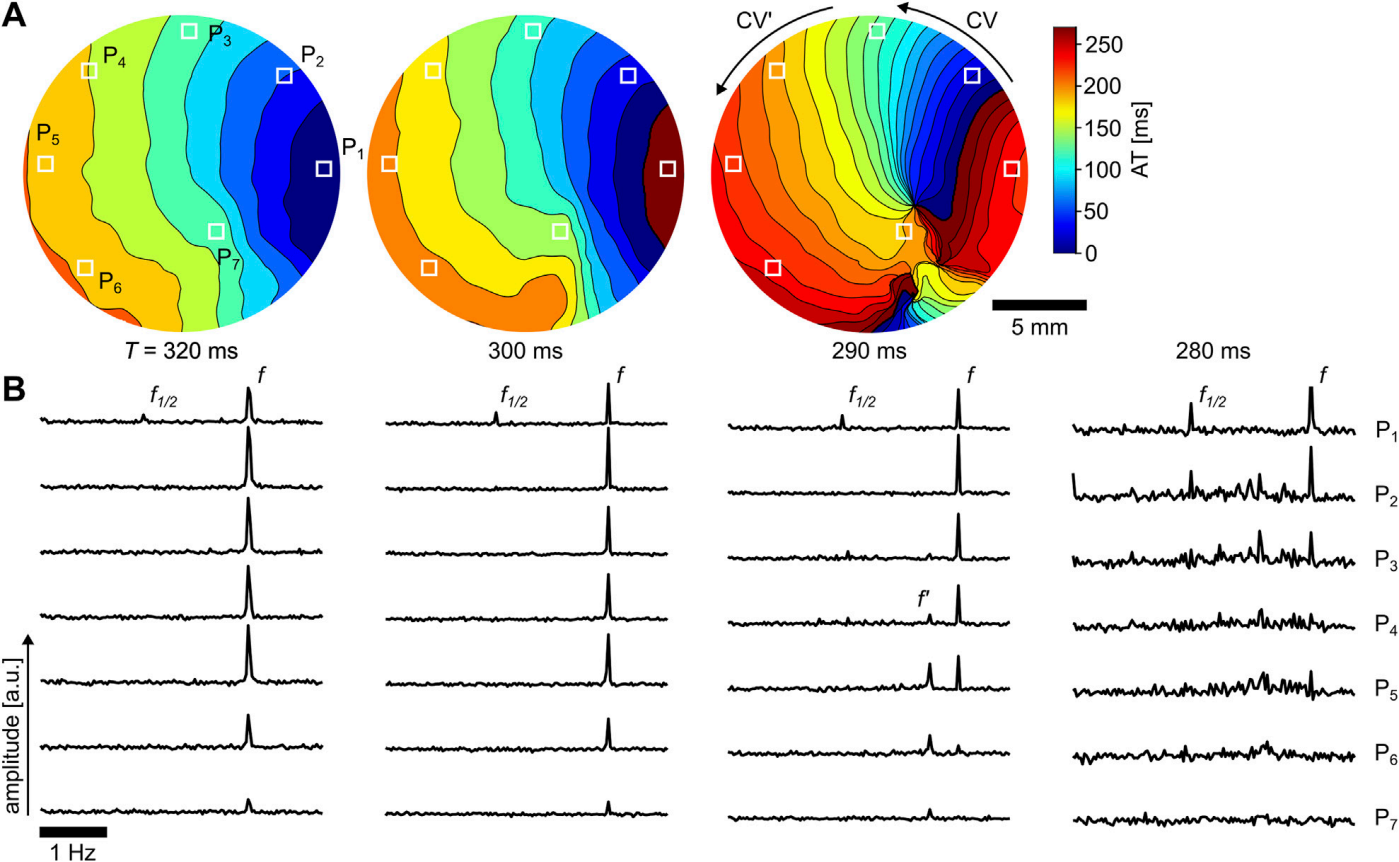
Initiation of spiral wave breakups from alternans. **(A)** ATMs for stimulation periods of T=320 ms (SDA), 300 ms (SDA), and 290 ms (spiral wave breaks). **(B)** Fourier spectra of the three ATMs illustrated in **(A)**, and the Fourier spectra of the spatiotemporal chaotic wave formation at T=280 ms. The spectra are obtained at the locations $P_{1}$ and $P_{7}$ , as indicated with white squares ( 15×15 pixels) at the first ATM in **(A)**. The Fourier spectra at T=290 ms (third column) show a frequency delay propagation of the paced wave from f≃3.6 Hz to $f^{\prime }≃3.1$ Hz, which corresponds to a ratio of 9 to 8. The arrows above the ATM indicate the change in the conduction velocity from CV to the slower CV ′ .

Activation time maps (ATMs) are used for the visualization of the transition to spiral waves at T=290 ms, where the tissue is no longer entrained by one or two underlying frequencies anymore but by more complex frequency distributions. Figure 7A shows the ATMs for T=320 ms, 300 ms, and 290 ms, depicting the transition from SDA to spiral waves in continuously paced tissues. In the lower right side of the tissue (around the location of $P_{7}$ ), a slowing of the CV can be observed as a narrowing of ATM isolines (black lines), which leads to a conduction block and the initiation of a pair of spiral waves when paced with T=290 ms. This slowing down of the CV can also be observed in the upper part of the tissue, as indicated with black arrows from CV to CV ′ . However, it does not lead to a conduction block. The observed pattern is stable as long as the tissue is electrically stimulated. Once the stimulation stops, no stable spiral wave remains. However, when stimulated with T=280 ms, the patterns become more chaotic, leading to a non-stationary pattern. Because of the non-stationary pattern, the ATM could not be applied for visualization. However, it is possible to visualize the Fourier spectra of the raw data, as shown in Figure 7B for the corresponding T shown in Figure 7A and T=280 . Seven spectra are shown from locations that are indicated by white squares in the first ATM of Figure 7A. The entrainment frequency $f=T^{-1}$ is indicated by the main peak. For T=290 ms, a second peak $f^{\prime }$ appears at the locations $P_{4}$ to $P_{7}$ , which indicates the occurrence of frequency-dependent delay propagation (FDP).

FDP is a spatiotemporal phenomenon where a single high-frequency source leads to more than one frequency domain within the tissue (Berenfeld et al., 2000). It is caused by tissue structurally induced delays of the propagating AP that may lead to fibrillation (Berenfeld et al., 2002). In the case shown in Figure 7 for T=290 ms, a frequency-lock ratio of 9:8 is observed in parts of the tissue as the stimulation frequency f≃3.6 Hz and the secondary frequency $f^{\prime }≃3.1$ Hz entrain stably. This spatially restricted calcium instability is a Wenckbach-like rhythm with a general frequency-lock ratio of N:M, i.e., M CTs are observed in response to N external stimuli (Bien et al., 2006). A further decrease in the stimulation period, i.e., increase in f , leads to spatiotemporal chaos (see Figure 7B, right panel). Although a stable period-2 alternans is observed at location $P_{1}$ , tissue further away from the pacing source exhibits chaotic wave conduction with non-stationary patterns.

### 3.5 Substrate-dependent initiation of alternans

**FIGURE 8:**
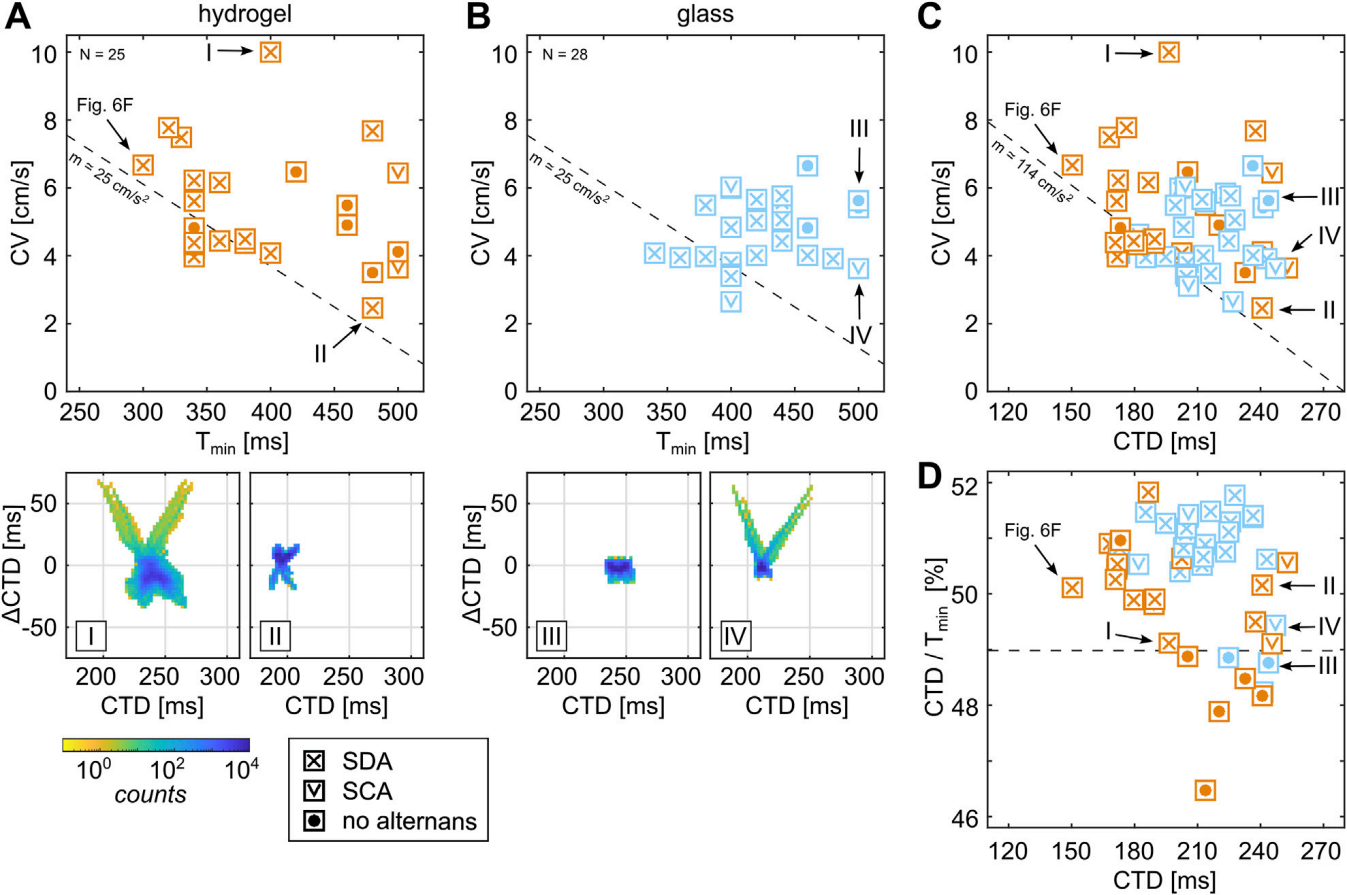
Comparison of the critical pacing period of cardiac tissues on hydrogel and glass. **(A, B)** CVs of entrained cardiac tissues paced with $T_{\text{min}}$ for hydrogel (orange) and glass (blue) substrates. The dashed lines indicate the virtual thresholds with a slope of $m≃25{\text{ cm/s}}^{2}$ . The lower panels show examples of probability phase maps that are indicated in the upper panels (I to IV). **(C)** CV as a function of the CTD. The dashed line indicates the virtual threshold with a slope of $m≃114{\text{ cm/s}}^{2}$ . **(D)** Ratio of CTD and $T_{\text{min}}$ as a function of CTD for both substrates—hydrogels (orange) and glass (blue). The dashed line indicates the minimum required ratio to observe alternans.

As the initiation of alternans is altered when tissues are cultured on ECMs that mimic the natural tissue environment, i.e., substrate rigidity is equal to the cell rigidity (Hörning et al., 2012a; 2013), we next spatially quantify and statistically compare the network physiological properties of calcium transient alternans on two different substrates—soft hydrogels ( E≃12 kPa, N = 25) and rigid glass ( E≃50 GPa, N = 28). Using the same pacing protocol, we determined the minimal pacing period $T_{\text{min}}$ that the tissues can be entrained with before the initiation of wave breakups, as exemplified in Figure 7. Only tissues that could be entrained with T≤500 ms were used to ensure comparability and reproducibility of the statistical outcome. Figure 8A, B show the CV at the respective $T_{\text{min}}$ for tissues grown on hydrogels and glass substrates. The three different observed dynamics are marked with ImgGF [d1], ImgGF [d2], and ImgGF [d3], referring to SDA, SCA, and normal wave conduction without the occurrence of alternans. The bottom panels illustrate four examples, which are marked by arrows in all other panels. For tissues cultured on hydrogel substrates, a virtual threshold can be identified where the CV negatively correlates with $T_{\text{min}}$ at a slope of approximately 25 cm/s^2^ (Figures 8A, B, dashed lines). This threshold functions as a visual aid only as there is not enough data to justify a numerical fit that defines the lower boundary of $T_{\text{min}}$ . This threshold does not apply well for glass; however, when comparing CV and CTD observed at $T_{\text{min}}$ , both hydrogels and glass samples show a common threshold with a slope of approximately 114 cm/s^2^ (Figure 8C, dashed line). Despite the mechano-regulative difference between the substrate conditions, a common electrophysiological relation between CV and CTD could be identified that marks the threshold of stable propagating waves and is independent of the substrate conditions. Data points above this line depict tissues with wave breakups that are most likely caused by tissue heterogeneities only.

Although normal wave conduction (ImgGF [d4]) is observed in the combination of larger $T_{\text{min}}$ and CVs in tissues cultured on glass substrates, a few tissues exhibit SDA and SCA at an even larger $T_{\text{min}}$ and larger CVs when cultured on hydrogels (Figure 8A, see also example I). Alternans forms when CTD has a certain ratio to T . Figure 8D shows the fraction CTD $/T_{\text{min}}$ as a function of CTD for both substrates—hydrogels (orange) and glass (blue). Tissues that exhibit SDA and SCA are located above 49% (black dashed line), while those that exhibit normal conduction are located below this line. So, alternans forms when the fraction CTD $/T_{\text{min}}$ is approximately 50%, where the alternation between longer and shorter CTDs balances out (see Figure 2A). However, one should keep in mind that CTD is the mean value of the tissue, so it is a rather crude quantifier and may be biased or insensitive to specific spatial areas within the tissue. Interestingly, one tissue was observed that shows perfectly normal wave conduction (ImgGF [d5]) despite a fraction CTD $/T_{\text{min}}$ of approximately 51%. This might indicate the existence of an unstable equilibrium state of the physiological properties or, contrarily, the alternation of the membrane potential while calcium transients are stable.

### 3.6 Substrate-dependent restitution and dispersion

**FIGURE 9:**
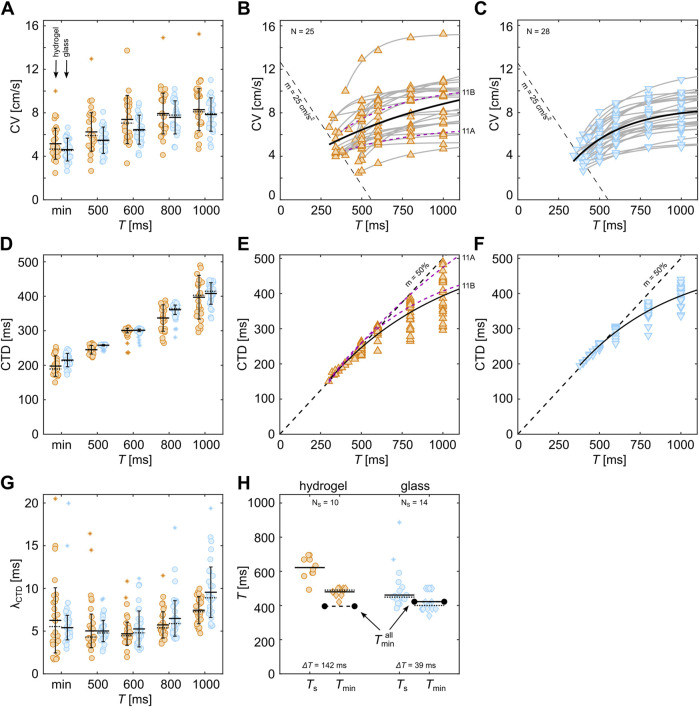
Comparison of restitution and dispersion properties of cardiac tissues on hydrogel and glass. **(A–C)** Comparison between restitution properties, **(D–G)** comparison between dispersion properties, and **(H)** comparison between spiral period $T_{\text{s}}$ (circles) and the respective $T_{\text{min}}$ (diamonds) between tissues on hydrogel (orange) and glass (blue). Horizontal solid and dotted lines indicate the statistical mean and median, respectively, and the error bars represent the standard deviation of the data. The asterisk markers depict outliers. $T_{\text{min}}^{\text{all}}$ (encircled dashed lines) shows the average $T_{\text{min}}$ of all observed data, independent of the emergents of stable spiral waves. ΔT indicates the difference between $T_{\text{s}}$ and $T_{\text{min}}$ .

Figure 9 shows the comparison between the restitution and dispersion properties of the tissues shown in Figure 8. Statistically, tissues on hydrogel show slightly higher CVs than those on glass substrates. This is more pronounced for lower pacing periods ( T≤600ms , Figure 9A). When comparing the dispersion curves of the individual tissues, a larger variability is observed for tissues on hydrogels (Figures 9B, C). Not only are there dispersion curves that could be classified as outliers, such as those with much smaller or larger CVs, but there is also larger variability among the individual slopes, as indicated by gray lines. The functional correlations are obtained through non-linear least squares fitting using a basic exponential function, as follows:

$$\text{F}\left(T\right)=\alpha +\beta \cdot \exp\left(-\frac{T}{\tau }\right),$$ (5)

where α defines the maximum CV for T→∞ and β and τ define the slope parameters of the exponential function. The solid black line indicates the all-in-all fit depending on the substrate type. Hydrogels show an approximately 50% lower α , i.e., maximum CV and a much flatter slope ( β and τ ) compared to tissues cultured on glass (see Table 1; Figures 9B, C). For the sake of completeness, the negative correlation for CV $\left(T_{\text{min}}\right)$ is indicated by the dashed black lines in Figures 9B, C, as shown in Figures 8A, B.

**TABLE 1:** Fitting parameter for restitution and dispersion curves of tissues cultured on hydrogels and glass substrates determined using Equation 5. The errors are the standard deviations (68% confidence intervals).

	Dispersion—CV vs. T			Restitution—CTD vs. T		
	α [cm/s]	β [cm/s]	τ [ms]	α [ms]	β [ms]	τ [ms]
Hydrogel	10.4 ± 3.2	9.0 ± 1.7	593 ± 640	547 ± 81	589 ± 37	742 ± 250
Glass	8.5 ± 0.6	15.1 ± 5.4	309 ± 109	549 ± 42	612 ± 15	686 ± 130

In contrast to the dispersion properties, the restitution properties show much less prominent variability (Figures 9D–F). The average CTDs obtained on hydrogels are comparable to glass but show a slightly larger variability. Figures 9E, F show the restitution curves without the fittings of the individual curves. Only the all-in-all-substrate-dependent fits (solid black lines) are shown. The latter culminates for smaller T to the slope of approximately 50% (dashed line), enabling a more accurate determination and comparison between the restitution properties of the two substrate conditions. The fitting parameter using the same basic exponential equation (Equation 5), as used for the dispersion fits, leads to very similar parameters (see Table 1). This indicates that the average CTD, i.e., electrophysiological properties of cardiac cells, does not significantly alter when cultured on soft or rigid substrates, unlike the dispersion properties (Figures 9A–C). So, the development of confluent tissues on soft or rigid substrates alters mainly cell-to-cell communication, which can be explained by the extended ability for cells to contract more efficiently on soft substrates, causing an increase in mechano-regulative dynamics during the developmental processes. This finding is in line with previous findings on the stability of spontaneous wave dynamics, where higher periodicity of beats was observed on substrates that match the muscle cell rigidity (Hörning et al., 2012a; 2013).

### 3.7 Substrate rigidity-dependent wave stability

Next, we investigate how the variability of CTDs in cells within tissues affects the generation of wave breakups and the formation of stable waves. Figure 9G illustrates this variability by calculating the standard deviation of the measured CTDs in each tissue $\left(\lambda_{\text{CTD}}\right)$ . The variability of CTDs in cells is lower for tissues on hydrogels for larger entrainment periods (T≥600 ms) despite comparable average CTDs on hydrogels and glass substrates (Figure 9D). A general trend toward lower variability can be observed when the interaction between subsequent waves increases. At approximately T=500 ms, a comparable mean $\lambda_{\text{CTD}}$ of approximately 5 ms can been observed. In the case of $\lambda_{\text{CTD}}\left(T_{\text{min}}\right)$ , hydrogels have a larger value, which is related to the lower average $T_{\text{min}}$ , referred to as $T_{\text{min}}^{\text{all}}$ from this point onward. Hydrogels and glass substrates exhibit $T_{\text{min}}^{\text{all}}$ values of 396 ms and 422 ms , respectively.

After the determination of $T_{\text{min}}$ for each sample, we intentionally tried to induce stable spiral waves using high-frequency stimulation $\left(T<T_{\text{min}}\right)$ . A lower probability of stable spiral wave initiation was observed on hydrogels, with 40% ( $N_{\text{S}}$ = 10 of 25), compared to glass substrates, with 50% ( $N_{\text{S}}$ = 14 of 28). Figure 9H shows the comparison of $T_{\text{min}}^{\text{all}}$ (encircled dashed lines), $T_{\text{min}}$ of the tissues that maintained stable spiral waves (diamonds), and the spiral wave period $T_{\text{S}}$ (circles). $T_{\text{S}}$ is comparable to $T_{\text{min}}$ on glass with an average difference of only ΔT=39 ms , while a significantly larger average difference of ΔT=142 ms is observed on hydrogels. This indicates that periodically entrained planar waves on glass tend to break up when reaching a critical pacing period, which is limited by the excitability of the tissue as wave-to-wave interactions do not permit lower wave periodicities. Contrarily to glass, stable spiral waves on hydrogel rotate at significantly larger periods. Due to their larger CVs (see Figure 9A) but comparable restitution properties, i.e., CTDs, the wave fronts of the spiral waves reach the still inhibitory wave tails faster and thus result in larger spatial spiral tip movements until a non-inhibitory wave tail is reached. This spiral tip movement is also known as meandering (Qu et al., 2000; Hussaini et al., 2023). Stable spiral waves stabilize only on tissues with larger $T_{\text{min}}$ compared to $T_{\text{min}}^{\text{all}}$ , which is the average critical period of all observed samples. This indicates the existence of even larger $T_{\text{S}}$ that could not be maintained at the 22-mm-sized cardiac tissues. In the case of glass, $T_{\text{min}}^{\text{all}}=422\text{ ms}$ falls almost perfectly on the average $T_{\text{min}}$ for stably induced spiral waves.In summary, these results indicate that cardiac tissues on hydrogels have a lower likelihood of forming stable spiral waves because of the larger dynamical range (lower $T_{\text{min}}^{\text{all}}$ ) that the tissues can be stably entrained with and the larger spatial spiral tip movements that require larger tissue sizes for stable initiation.

**FIGURE 10:**
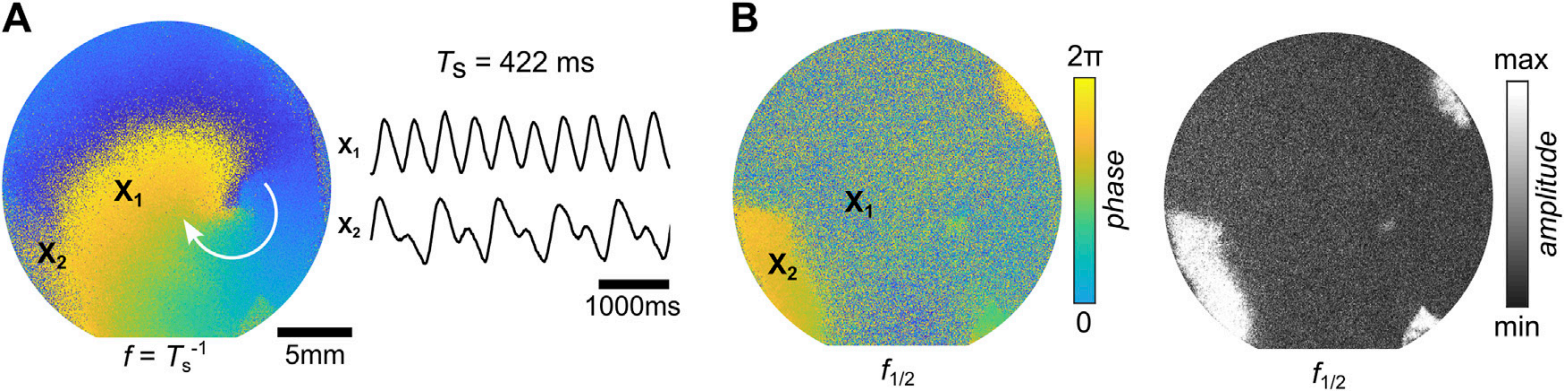
SCA in a spiral wave. **(A)** Stable spiral wave in cardiac tissue on a glass substrate that exhibits SCA and two calcium transient traces of normal wave conduction (top, $\times_{1}$ ) and alternans (bottom, $\times_{2}$ ). **(B)** Respective phase and amplitude for the frequencies f and $f_{1/2}$ obtained by FFI.

In very few tissues (N = 1), the formation of stable alternans was observed. Figure 10 shows the FFI analysis of this spiral. The Fourier phase map of the frequencies $f=T_{\text{s}}^{-1}$ is illustrated in Figure 10A. Although the stable spiral wave is visible in the phase map of f , spatial regions that exhibit alternans can be identified as the phase-synchronized and high-amplitude areas in $f_{1\text{/}2}$ (Figure 10B). For comparison, two calcium transient traces of normal wave conduction (top, $\times_{1}$ ) and alternans (bottom, $\times_{2}$ ) are shown in Figure 10A. This tissue partly shows SCA. The corresponding probability phase map is shown as example IV in Figure 8B. SDA dynamics are also possible in free and obstacle-bound stable spiral waves (Hörning et al., 2017; Kim et al., 2007). However, SDA-exhibited spirals have only been observed on glass substrates so far, which may indicate the influence of the reduced contractibility in cardiac tissues cultured on rigid glass substrates. Furthermore, the spiral wave observed in this study shows SCA on the periphery only, while more complex dynamics are observed when the spiral core interacts with the formed nodal lines (Hörning et al., 2017; Kim et al., 2007), as seen in the paced spiral wave illustrated in Figure 7A (right panel). This phenomenon has been theoretically discussed and explained by the occurrence of so-called supernormal conduction, also known as anomalous dispersion, in generic excitable media (Echebarria et al., 2011).

## 4 Conclusion

We have introduced a simple but effective way to identify and quantify alternans in bioengineered cardiac tissues by introducing probability maps of CTD and Δ CTD that were extracted by an improved signal oversampling technique. We discussed the development of wave dynamics with decreasing stimulation periods from normal conduction to SDA and its destabilization, which can lead to the formation of stable spiral waves. Although these dynamics are observed in tissues cultured on soft hydrogels and rigid glass, we have statistically shown that the dispersion properties differ most for the two different substrate conditions. Restitution properties, on the other hand, remain comparable and only differ in the variation of cells within the tissues, which might be explained by mechano-regulative processes during tissue development. We provide the parameterization of the dispersion and restitution curves obtained from both substrate conditions (see Table 1), which may serve as input for further *in silico* investigations, similar to those performed by Loppini et al. (2022).

**FIGURE 11:**
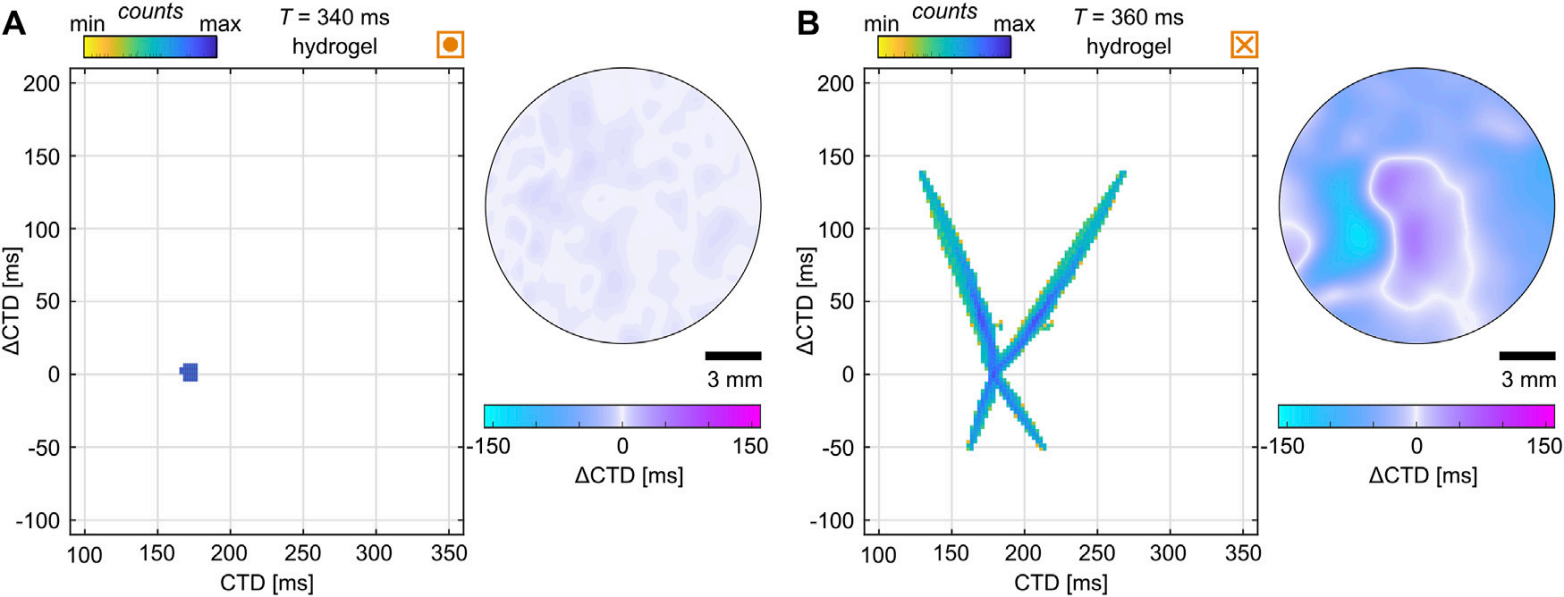
Examples of similar electrophysiological properties but different dynamics. **(A, B)** Probability phase maps (left) and ΔCTD maps (right) of cardiac tissues cultured on hydrogels. **(A)** Tissue with normal wave conduction and **(B)** tissue with SDA. The respective restitution and dispersion curves are highlighted by dashed purple lines in Figures 9B, E.

The statistical analysis of the critical pacing period revealed a common substrate-independent threshold between CTD and CV. As it remains difficult to classify the quality of bioengineered tissues, the general validity remains to be shown for naturally grown tissues and more physiologically relevant temperature ranges as we investigated hypothermic conditions (room temperature) only. We identified common electrophysiological properties when alternans forms (Figure 8D); however, despite very similar electrophysiological and ECM properties, there are cases that defy the odds and do not show alternans. Figure 11A shows such an example where no alternans was detected. For comparison, a tissue that exhibits very expansive SDA at very similar CVs, CTDs, and substrate conditions is shown in Figure 11B. Those two tissues illustrate that there are still unknown properties that may cause alternans. Such a property in the ultrastructure of the tissue could be related to recent findings, such as the extraction of ultrastructure from *ex vivo* canine ventricles to recover SDA dynamics *in silico* (Loppini et al., 2022) or the difference in the restitution and dispersion properties (see dashed purple lines in Figures 9B, E). Thus, further investigation is also needed on a theoretical level to study potential instabilities in the intracellular calcium cycling.

The substrate-depending dynamics of CTs revealed their influence on the stability of spiral waves. Myocardial scars have altered structural and mechanical properties compared to healthy tissue (Richardson et al., 2015; Münch and Abdelilah-Seyfried, 2021), which can facilitate the formation and stabilization of spiral waves in cardiac tissue (Connolly and Bishop, 2016; Song, 2023). In line with this, we showed that the enhanced mechanical contractility of cells on soft substrates, which mimic native extracellular matrix properties, lowers the likelihood of forming stable spiral waves; this is due to increased wave stability and change in excitability, leading to larger spiral tip trajectories and a greater chance of self-termination at the boundary of the tissue. Interestingly, reduced contractility on glass substrates seems to promote the formation of SCA and SDA in spiral waves, as observed in this study (see Figure 11) and in Hörning et al. (2017). We have not observed stable spiral waves with alternans in tissues cultured on soft hydrogels.

Although this study focuses on the comparison of CTs in tissues cultured on soft and rigid substrates, the introduced analysis approach can also be potentially applied to *ex vivo* experiments to visualize alternans dynamics (Berenfeld et al., 2000; Loppini et al., 2022). Additionally, theoretical consideration would be useful to further understand the role of clinically relevant aspects, such as the influence of ectopic beats, the effect of the bidirectional coupling between voltage and calcium signaling, and the role of the electronic coupling between cells (Weiss et al., 2006; Qu and Weiss, 2023). The application to alternans observed in the AP and contraction amplitude remains to be shown, but it might reveal auspicious and useful biomedical insights, especially when using simultaneous records of APs, CTs, and contraction waves in cardiac tissue (Kong et al., 2003; Wang et al., 2014; Liu et al., 2023; Crispino et al., 2024). This would be especially important to further discuss the dynamics and differences of electrical (Courtemanche et al., 1993; Echebarria and Karma, 2002; Bauer et al., 2007) and calcium-driven alternans (Shiferaw et al., 2005; Sato et al., 2006; Skardal et al., 2014) on a theoretical level as only the combination of both may suffice to fully understand alternans instability and the role of the ECM.

## Data Availability

The raw data supporting the conclusions of this article will be made available by the authors, without undue reservation.
