# Supplementary material for: Native mechano-regulative matrix properties stabilize alternans dynamics and reduce spiral wave stabilization in cardiac tissue
Source: Front Netw Physiol. 2024 Sep 24;4:1443156. doi: 10.3389/fnetp.2024.1443156 (PMC11458432; doi:10.3389/fnetp.2024.1443156)
Supplement: Supplementary file 1 [file DataSheet1.PDF]

Supplementary Material

1 SUPPLEMENTARY TABLES AND FIGURES

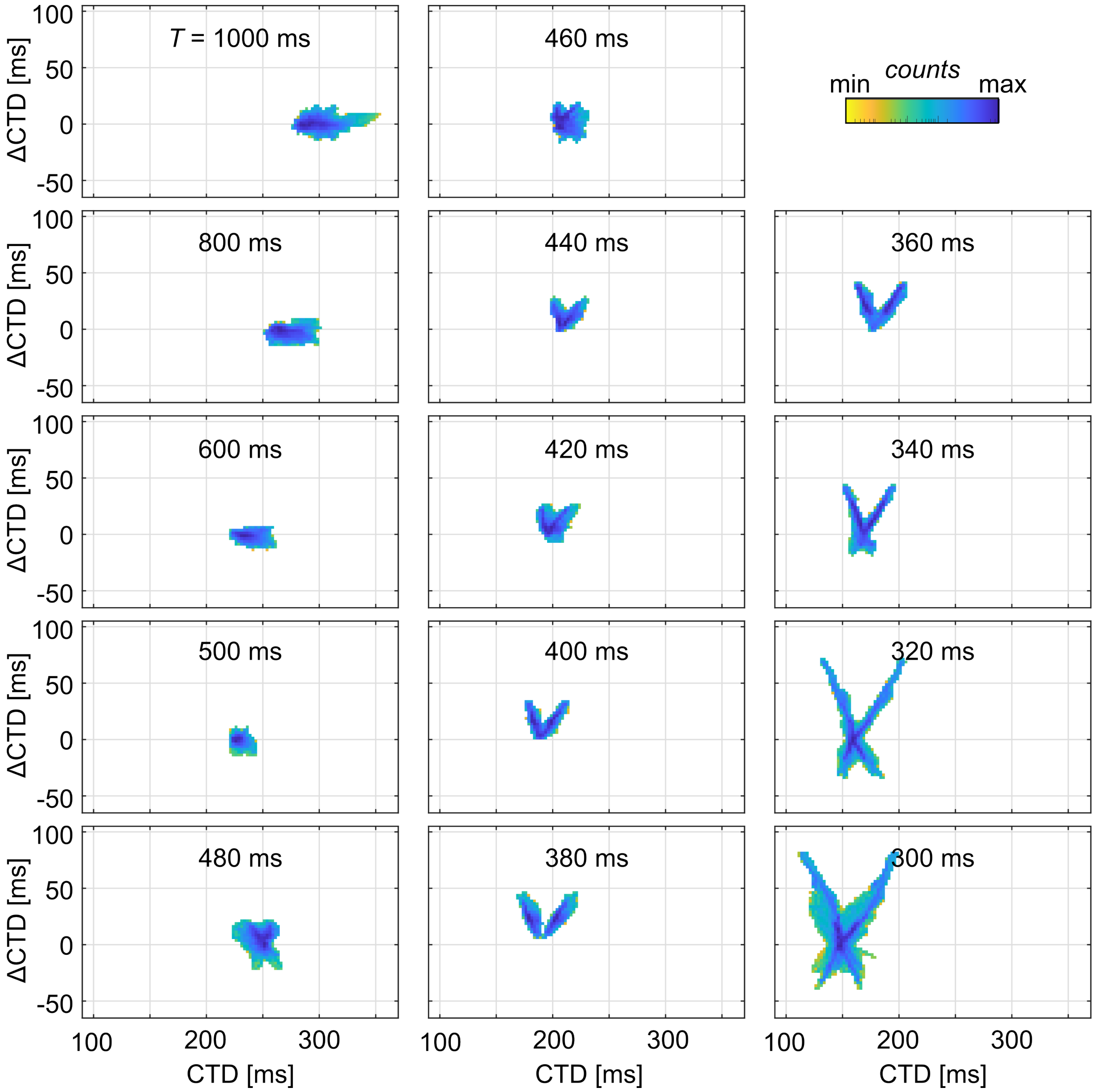

Figure S1. Probability phase maps for all stimulation periods. Shown is normal conduction from $T = 1000$ to $T = 460$ ms, SCA from $T = 440$ ms to $T = 360$ ms, and SDA from $T = 340$ ms to $T = 300$ ms.
